# The regional impact and control strategies of schistosomiasis in Rwanda, Tanzania, and Uganda: a narrative review

**DOI:** 10.1097/MS9.0000000000004584

**Published:** 2025-12-11

**Authors:** Niyibizi Julius, Run (Sherry) Wang

**Affiliations:** a Pharmacist, Kigali City, Rwanda; b Southern Medical University, China; c Southern Medical University Institute for Global Health (SIGHT), Dermatology Hospital of Southern Medical University (SMU), Guangzhou, China; d Acacia Lab for Implementation Science, School of Health Management and Dermatology Hospital, Southern Medical University, Guangzhou, China; e Center for World Health Organization Studies and Department of Health Management, School of Health Management of Southern Medical University, Guangzhou, China

**Keywords:** One Health, climate change, mass drug administration, schistosomiasis, WASH, zoonotic transmission

## Abstract

**Background:**

Schistosomiasis remains a major public health problem in East Africa, particularly Rwanda, Tanzania, and Uganda, driven by inadequate sanitation, unsafe water contact, and snail intermediate hosts.

**Objective:**

This review summarizes recent epidemiological information and reviews schistosomiasis control efforts in East Africa to highlight the achievements, challenges, and pathways to elimination.

**Methods:**

We reviewed 37 recent studies on prevalence, morbidity, and intervention, including mass drug administration (MDA), water sanitation and hygiene (WASH) measures, snail control, and health education. Emphasizing inter-sectoral approaches and One Health.

**Results:**

Findings vary across regions, with 15.9% *S. haematobium* in the Simiyu Region in Tanzania, to declining but persistent *S. mansoni* in Uganda, while Rwanda has succeeded in lowering the prevalence of schistosomiasis using combined interventions. MDA and WASH have lessened morbidity in school-aged children; however, challenges persist, including low-sensitivity diagnostics, climate-mediated snail expansion, zoonotic aspects, and gender inequalities. Interventions for preschool-aged children and adolescent girls are often excluded, undermining control efforts.

**Conclusion:**

Elimination of schistosomiasis requires improved diagnostics, climate-adapted snail control, equitable MDA coverage, and a One Health approach. Achieving WHO’s 2030 targets demand innovation, political commitment, and equitable allocation of resources to address this public burden.

## Introduction

Schistosomiasis, an infection with trematode worms of the genus *Schistosoma*, is endemic in East Africa, posing a significant public health challenge. Its preventable nature makes its persistent burden particularly striking. The disease is acquired by contact with freshwater containing infected snails and occurs in two main forms in the area: urinary (*Schistosoma haematobium*) and intestinal *(S. mansoni*) schistosomiasis. East Africa contains the lakes and rivers where water bodies provide ideal habitats for snail intermediate hosts, complicating control efforts due to their prevalence in marshy and wet terrains^[1,2]^. The need for new interventions, including vaccines, is increasing since praziquantel, the primary treatment, could not control reinfection^[3]^.

**HIGHLIGHTS**

- Schistosomiasis prevalence varies across East Africa, with Rwanda showing significant progress through integrated control, while Tanzania and Uganda face persistent challenges.
- Mass drug administration (MDA) and WASH interventions reduce morbidity, but gaps in coverage for preschool-aged children and adolescent girls hinder elimination efforts.
- Climate change exacerbates transmission by altering snail habitats, necessitating adaptive snail control and One Health strategies.
- Low-sensitivity diagnostics like Kato–Katz limit accurate prevalence assessment; advanced tools like SHERLOCK show promise but require rural adaptation.
- Gender disparities and zoonotic transmission highlight the need for inclusive, ecosystem-based approaches to achieve WHO’s 2030 elimination targets

Schistosomiasis is a disease of poverty, which is known to flourish where sanitation is inadequate and access to clean water is restricted, putting the rural and marginalized at greater risk^[4]^. This has a disproportionate effect on children, particularly school-aged children, who are exposed to water for longer periods while playing or performing chores^[1]^. Given its chronic nature, the disease not only leads to acute illness but also results in long-term outcome, including liver damage and bladder cancer, thereby underscoring the critical importance of effective control measures^[5]^. Schistosomiasis imposes a heavy disease burden in East Africa, with chronic infections leading to severe long-term consequences such as hepatic fibrosis, bladder cancer, and stunted growth in children^[5]^, affecting up to 20–30% of infected school-aged youth through reduced productivity and school attendance^[6]^. Prevalence remains stubbornly high in hotspots like Tanzania’s Simiyu Region (15.9% for *S. haematobium*)^[7]^ and Uganda’s Lango area^[8]^, despite regional declines^[9]^, while economic costs; including household out-of-pocket expenses for treatment and lost wages exacerbate poverty cycles, straining under-resourced health systems^[4]^ and underscoring the urgent need for this review to map pathways to elimination.

Despite progress in reducing prevalence in some countries, ongoing transmission and severe outcomes such as liver damage and bladder cancer underscore the need for this review, given the few prior studies on regional challenges and successes^[8,10]^. It highlights the need for collaborative, innovative strategies to achieve elimination. This article complies with the TITAN 2025 guidelines repressing the reporting and use of AI^[11]^.

## Methodology

A structured narrative reviewed and data collection were performed from 37 peer-reviewed articles published from 2004 to 2025 on the epidemiology, morbidity, and control of schistosomiasis in Uganda, Tanzania, and Rwanda, selected due to their high disease burden and diverse control strategies, justifying a focused analysis. This 21-year period was chosen to capture long-term trends and recent advancements in control measures. The articles were retrieved by using the following search terms: schistosomiasis, mass drug administration, WASH, and One Health in the databases of PubMed, Web of Science, and Scopus with these interventions selected as they represent the primary, evidence-based strategies for schistosomiasis control. As this was a narrative review rather than a systematic review, the retrieval process was not designed for exhaustive coverage but for comprehensive understanding through critical integration of relevant evidence. The searches returned a larger pool of records across the three databases, but after screening titles and abstracts for relevance and applying the inclusion and exclusion criteria, only 37 peer-reviewed articles published between 2004 and 2025 were retained for analysis. Multiple rounds of screening were conducted to ensure that included studies directly addressed epidemiology, morbidity, or control interventions in Uganda, Tanzania, and Rwanda. Grey literature, including WHO technical reports and country-specific health reports, was also considered to supplement peer-reviewed data. The inclusion criteria included all studies that dealt with prevalence, morbidity, or some form of intervention (MDA, WASH, snail control, or health education) in the mentioned countries. The exclusion criteria were other East African countries that do not have primary data or studies based on secondary data. Descriptive quantitative data of key outcomes (e.g., prevalence ranges, MDA coverage rates, reduction in morbidity) were summarized narratively. The data on the control of schistosomiasis were analyzed qualitatively to identify trends, successes, failures, and gaps in control. A formal meta-analysis was not conducted due to heterogeneity in study populations, interventions, and outcomes, but the narrative synthesis was structured to allow clear comparison of regional trends and control strategies.

## Disease burden in East Africa

### Prevalence

The distribution of schistosomiasis in Uganda, Rwanda, and Tanzania is heterogeneous and is influenced by geographic, ecological, and social factors. A survey of 21 primary schools in Chikwawa District, southern Malawi, reported a urinary schistosomiasis prevalence of 35.0% and an intestinal schistosomiasis prevalence of 1.9%^[1]^. Although Malawi is not the primary focus of this study, it provides relevant context for regional comparisons. A 15.9% prevalence of *S. haematobium* was recorded in high-school students in the Simiyu Region of Tanzania^[7]^. In the Lango region, Uganda, the prevalence of *S. haematobium* has decreased over decades, yet *S. mansoni* remains a persistent challenge^[8]^.

Countrywide data for Tanzania, covering 2.5–4 million schoolchildren, indicated that most areas had a prevalence of >10%; hotspots, of more than 30%, were found in the northwest, southwest, and southeast^[12]^. Meanwhile, Rwanda has made progress, with transmission decreasing through years of control efforts, though some foci remain^[10]^. During a nationwide remapping exercise in Rwanda with POC-CCA as a rapid test for *S. mansoni*, it was observed that the prevalence of *S. mansoni* is greatly underestimated using the routine and traditional Kato–Katz method, which once again demonstrates the continued problems in achieving reliable detection of this parasite^[13]^. These variations suggest that schistosomiasis may manifest as a local epidemic rather than a uniform disease. Seasonal dynamics also play a role. *S. haematobium* transmission in Tanzania’s Simiyu Region is elevated during the rainy season, which is a function of snail abundance^[14]^.

Preschool-aged children (PSAC) are easy to overlook, yet one study near Lake Victoria in Uganda revealed a high *S. mansoni* prevalence among 1- to 5-year-olds, indicating that they are a hidden reservoir^[15]^. This result highlights the importance of including PSAC in surveillance for a complete picture of the epidemiology. Co-infections, such as with malaria and schistosomiasis in Malawi, are reported at 5.5% and further complicate the epidemiology^[1]^. Despite a decrease in prevalence in sub-Saharan Africa between 2000–2010 (23.0%) and 2015–2019 (9.6%), East Africa remains a schistosomiasis endemic region^[9]^.

### Morbidity

Morbidity from schistosomiasis is insidious and slowly progressive, often accumulating over time. Urinary Schistosomiasis can result in hematuria, bladder lesions, and cancer, whereas intestinal Schistosomiasis causes abdominalgia, diarrhea, and hepatic fibrosis^[5]^. In Uganda, *S. mansoni*-associated periportal fibrosis is a serious issue, with prevalence rates of 10–15% in endemic areas, and its incidence is not always consistent with the level of current infection, suggesting that other risk factors, including HIV or chronic hepatitis, may contribute^[16]^. This inconsistency indicates an ongoing challenge in understanding multiple determinants of morbidity.

Children are affected in the most apparent ways. In Malawi, schools that exceed the national average for urinary schistosomiasis prevalence reflect its heavy impact on young bodies^[1]^. Chronic infection can also affect stunted growth, impaired cognitive development, and decreased school attendance^[6]^, with studies estimating 20–30% of infected children experiencing growth deficits. The “Worm Years” prevalence measure of morbidity in PSAC shows that there can be a gain by targeting these children for MDA even if elimination is not achieved^[6]^.

Cross-species transmission adds another complication. In Uganda, *S. mansoni* infections in chimpanzees and domestic animals indicate the presence of zoonotic transmission that may drive infection among humans^[17,18]^. This zoonotic dimension indicates the need for a One Health strategy integrating human, animal, and environmental health. Morbidity indicators should also be refined. Heavy intensity infections are currently the focus of attention, and the present-day sensitivity is poor; a measure of overall prevalence may more accurately reflect the public health burden^[5]^. This alteration in metrics may enhance control tactics by capturing the entire illness load.

### Socioeconomic impacts

Schistosomiasis is not only harmful to the body, but also a poverty trap for communities. In the Ukerewe District of Tanzania, an intervention on hygiene and sanitation (PHAST) reduced prevalence by 15–20% and increased household wealth, demonstrating a proven socioeconomic success^[4]^. This intervention underscores the potential for health programs to yield economic benefits.

Chronic schistosomiasis infections contribute to the perpetuation of poverty through impaired physical growth, cognitive development and reduced schooling in infected children who constitute 20–30% with severe growth retardation^[6]^. The net effect of these outcomes is a loss in long term productivity and earning potential with affected families being unable to escape the cycle of poverty while hindering wider community capacity building

The economic cost also is found in the suppression of a vaccine response. Immune responses to vaccines such as BCG may be suppressed following *S. mansoni* infection in Ugandan children; however, there is inconsistent evidence for successful reversion of praziquantel (PZQ) treatment to restore this^[19]^. This interaction proves and suggests that schistosomiasis control is integral to much broader public health objectives.

### Control strategies

Throughout Rwanda, Uganda, and Tanzania, a variety of approaches have been tested to control schistosomiasis and these have yielded mixed results. These initiatives are largely focused around mass drug administration (MDA), snail control, WASH interventions, and health education, which have their limitations and strengths.

### Mass Drug Administration

Mass Drug Administration (MDA) with praziquantel, which is safe and efficacious, remains the mainstay of schistosomiasis control^[20]^. MDA has led to a marked reduction in prevalence in sub-Saharan Africa among school-aged children as the main target^[9]^. The experience of Uganda is instructive: community-level MDA rather than district-wide units enhances targeting accuracy and minimizes the wastage of drugs^[21]^. This enhanced approach boosts intervention efficiency. Despite all these limitations of PZQ, including low solubility, fast metabolism, and emerging drug resistance, this clearly showed the hope for alternative treatments and delivery systems^[3]^. Intensive praziquantel treatment in Uganda led to decreased *S. mansoni* infection intensity with little effect on vaccine-associated responses, other than enhancing BCG-vaccine specific cellular responses^[19]^.

Rwanda’s model, which combines MDA with diagnostics and partnership, has rapidly expanded coverage and moved toward elimination^[10]^. Incorporating PSAC in MDA, as explored in Uganda, leads to a reduction in morbidity but is not always sufficient to push the balance towards EPHP^[6]^. This indicates that MDA alone cannot be sufficient enough for elimination. The failure of Tanzania to include adolescents in MDA campaigns leaves a reservoir for continued transmission, and this is a miss^[7]^. These gaps can be overcome by the 2022 WHO guidelines, which increase MDA to all ages over 2 years and reduce prevalence thresholds^[22]^. These guidelines provide the potential to reduce transmission gaps.

The WHO outlines phases of schistosomiasis control, beginning from the initial control program to post-elimination surveillance, highlighting distinct strategic priorities relevant to intervention areas in East Africa^[23]^.

### Snail control

The control of snails, intermediate hosts (in the *Bulinus* and *Biomphalaria* species), is another way to interrupt transmission. Mathematical models indicate that the introduction of competitive or predatory snails can reduce the numbers of host snails, and they may eliminate the disease (although competition must be very strong)^[24]^. In Cameroon, residual transmission in snails highlights the importance of snail control in addition to MDA^[25]^. There is seasonal variation in snail densities in the Simiyu Region, Tanzania, and accumulation of schistosome cercariae in the sample, with greater cercarial shedding during the rainy season that would require timing of interventions^[14]^. A test-treat-track-test-treat (5T) study in Pemba Island, Tanzania, succeeded in keeping *S. haematobium* prevalence low by focusing only on infected individuals and proved to be a cost-effective option for low-prevalence areas, but the long-term effect is not clear yet^[26]^.

Snail control is promising but carries risks, as only weak competitor snails may fail to survive intense competition^[27]^. Studies on Lake Albert, Uganda, demonstrate *Biomphalaria stanleyi* as a primary intermediate host and propose snail control as a complimentary intervention to MDA^[2]^. However, environmental changes, such as changing water bodies, are difficult in irrigation-dependent areas^[1]^. These challenges highlight the need for integrated environmental management.

### Water, Sanitation, and Hygiene interventions

Water, Sanitation, and Hygiene (WASH) initiatives seek to minimize water exposure and the habitats of snails. Rwanda’s infusing WASH into MDA also changed the game, which has addressed the socio-environmental drivers^[10]^. In Tanzania, the PHAST intervention not just generated a reduction in prevalence but also had a “positive return” in terms of increased household wealth, a win-win^[4]^. WASH necessitates infrastructure, and rural East Africa frequently does not have it. For example, in Malawi, irrigation schemes provide replacement breeding sites for snails, offsetting WASH interventions^[1]^.

Gender inequities persist in WASH programs, as the roles of women in water collection are not acknowledged in behavioral change campaigns^[7]^. This oversight represents a significant gap in WASH intervention. Climate change makes matters worse, with paths of rainfall changing the dynamics of water bodies^[24]^. WASH implementation faces a high logistical challenge.

### Health education

Health education promotes behavior changes, such as avoiding dirty water. Tanzania’s research has shown that the positive attitudes towards schistosomiasis increase the risk, indicating that the message of education has to be addressed, including the cultural behavior^[7]^. Community mobilization through the PHAST approach in Uganda has empowered the people, and poverty has fallen since, as has prevalence^[4]^. Micro-epidemiology work in Zanzibar connects education with particular risk behaviors (such as swimming in infected streams)^[28]^.

Education does best when combined with MDA or WASH, but independent campaigns tend to fizzle^[20]^. Ignoring the need for direct community resources hinders standalone education campaigns. However, combined education on One Health and environmental management approaches, like in Rwanda, offers promising frameworks^[10]^.

Table 1 summarizes the control strategies implemented in Rwanda, Tanzania, and Uganda, alongside the key challenges hindering their effectiveness.

**Table 1:** Schistosomiasis control strategies and challenges in Rwanda, Tanzania, and Uganda

Country	Control strategies	Key challenges
Rwanda	Mass Drug Administration (MDA), Water, Sanitation, and Hygiene (WASH), and snail control; One Health approach; elimination-focused.	Diagnostic gaps; climate-driven snail expansion; resource sustainability.
Tanzania	Mass Drug Administration (MDA) (excludes adolescents); Participatory Hygiene and Sanitation Transformation (PHAST) for prevalence and poverty reduction; snail control [test-treat-track-test-treat (5T)]; health education.	Gender exclusion; cultural acceptance; seasonal transmission; adolescent reservoirs.
Uganda	Community-level Mass Drug Administration (MDA); snail control; One Health for zoonotic cycles; Preschool-Aged Children (PSAC) inclusion; Geographical Information Systems (GIS) targeting.	Zoonotic transmission; diagnostic limitations; competing health priorities; poverty-driven exposure.

### The problems and gaps in schistosomiasis control

Despite progress in the control of schistosomiasis in East Africa, the pathway to elimination still faces significant barriers like climate change, diagnostic limitations, gender inequalities, zoonotic transmission, and implementation challenges.

Significant progress has been made, but elimination remains challenging due to multiple persistent barriers.

### Climate change impacts

Climate change is another spanner, as seasonal spikes in transmission are moved around by temperature and rainfall changes^[27]^. Climate change significantly alters schistosomiasis transmission dynamics through directly affecting snail population and cercarial shedding. In the Simiyu Region, of Tanzania, the transmission of *Schistosoma haematobium* peaked during the rainy season^[14]^. A modelling study in Zimbabwe (not East Africa, but with similar ecological conditions) is informative; 26 environmental covariates, including precipitation and temperature, were found to shape regional differences in prevalence, likely a dynamic that also applies in Tanzania or Uganda^[29]^. Irrigation associated with the Shire Valley Transformation Program in Malawi is leading to the creation of new snail habitats, thus increasing the risks of transmission^[1]^.

Climate disease interaction poses a significant challenge; models explain how variability of the monthly amount of rain acts to increase the abundance of snails and that interventions like snail control or MDA require an accurate targeting^[24]^. Yet most programs don’t systematically include climate data, which seems to me like a lost opportunity. One Health strategies may include weather forecasts to predict transmission peaks, such as in Rwanda^[10]^, although usually they are not a feature of such strategies. The vagaries of climate change also raise questions about cost. Changing irrigation systems or expanding the activities of snail control in response to changing patterns of rainfall are costly, raising questions about feasibility in resource constrained settings.

### Diagnostic limitations

Diagnostics are the cornerstone of schistosomiasis control, and yet the present tools are failing us in small but crucial ways. Both the Kato–Katz for *S. mansoni* and urine filtration for *S. haematobium* fare poorly in low prevalence settings, missing low intensity infections that contribute to transmission^[30]^. Since the parasite burden is lower in Formerly Endemic Implementation (FoEI) areas than Persistent Hotspot Districts (PD) areas, which affects test accuracy as an infection becomes lighter, our data suggest that the POC-CCA tests also captured the presence of Schistosoma in previously untreated school-aged children who had tested negative with Kato–Katz with 95.6% sensitivity. This diagnostic gap hinders accurate assessment of control progress. A similar breakthrough is the CRISPR/Cas13a-based SHERLOCK assay, which is 93–100% concordant with qPCR for *S. japonicum* and more sensitive than qPCR for *S. mansoni*, detecting infection as early as 3 weeks post-exposure^[31]^. But there is a catch: SHERLOCK relies on lab equipment, something not commonly available at rural clinics in Uganda or Tanzania.

Haemastix™ reagent strips for micro-hematuria is a low-cost and practical alternative in Zanzibar, costing £0.20 per test and showing high sensitivity and specificity^[32]^. This affordability enables schools to implement testing cost effectively. However, diagnostics should move toward prevalence assessment and not solely the measurement of heavy-intensity infections, as the current EPHP by infection as a public health problem definition is obsolete^[5]^. In Uganda, controlled human infection model (CHI models) highlights the need for diagnostic support for vaccine trials beyond case detection^[33]^. The field is entering a transformative phase in diagnostics, though scaling these tools in remote areas remains logistically challenging.

### Gender disparities

Women and girls face higher exposure risks due to water collection and washing duties but are often excluded from control programs^[7]^. In the Simiyu Region of Tanzania, adolescents (predominantly girls) are not recipients of MDA and as such serve as a reservoir for onward transmission^[7]^. This systematic exclusion undermines control effort.

Cultural barriers contribute to the problem such as stigma attached to urinary symptoms which lead to a delay in seeking treatment by women^[7]^. Tanzania’s PHAST intervention, which empowered women and alleviated poverty, demonstrates what can be achieved when sub-community involvement is inclusive^[4]^. However, gender-based strategies, women-led education, or targeted MDA for female adolescents could close this gap, and the science about them are underrepresented from the literature and requires prioritization.

### Zoonotic and interspecies transmission

The zoonotic transmission complicates control efforts. In Uganda, the occurrence of *S. mansoni* infections in chimpanzees and livestock suggests animals sustain human transmission^[17,18]^. Such zoonotic cycle limits MDA’s effectiveness^[17]^. This One Health challenge necessitates an ecosystem wide approach.

The schistosomiasis control program in Tanzania unexpectedly decreased the prevalence of *Taenia solium* demonstrating the interconnectedness of parasite control programs^[34]^. The way Rwanda is employing the One Health framework (combining human, animal, and environmental approaches) is an example that adds to the debate^[10]^. However, scaling up such approaches is complex. Veterinary MDA or livestock management is believed to disrupt zoonotic cycles but cross-sectoral affair remains challenging.

### Implementation and resource placement

Implementation obstacles are a key barrier to the effective control of schistosomiasis. The use of districts as MDA units in Uganda has led to misclassification of community-level risk, over- or under-treatment, and praziquantel wastage^[21]^. Transferring the units to community level makes for a more precise unit, but requires better data and logistics^[21]^. From Cameroon’s experience, it is a shot across the bow: even MDA, WASH, and education, residual transmission in snail populations remains^[25]^. To address these challenges better funding and international cooperation is needed to achieve the WHO 2030 NTD Roadmap targets, especially on new diagnostics, drugs, and snail control technology^[23]^.

The allocation of resources remains a barrier. Schistosomiasis is a contending infection with malaria, HIV, and other priorities and resources are often withdrawn before elimination is achieved^[35]^. The support of the Bill and Melinda Gates Foundation through the projects in Uganda and Tanzania has provided lifelines, but the sustainability of the gains made is fragile^[35]^. Geographical information systems (GIS) could help with decisions on where high-risk areas exist with respect to schistosomiasis in Uganda, yet they are not used to their full potential^[36]^. Sustained political commitment is critical to prioritize this often-overlooked disease.

### A social and behavioral obstacle, the prevalence of HCV maintains itself within low socioeconomic, urban, and intravenous drug user communities and public access sites

The influence of human behavior and socioeconomic pressures add an extra complexity. Poverty leads to water contact as people depend on polluted rivers for fishing, bathing, or laundering, as in Zanzibar^[28]^. Behavior changes interventions from health education may work, but stand-alone campaigns die away if there are no MDA or WASH interventions to support them^[20]^. The PHAST intervention in Tanzania achieved prevalence and poverty reduction, but upscaling community-based models is resource-demanding^[4]^.

Apart from that, there are also barriers such as behavioral resistance. In Tanzania, positive attitudes towards schistosomiasis, such as accepting it to be part of routine life, are a proxy for risk, meaning education also needs to challenge deeply held norms^[7]^. Addressing cultural acceptance of schistosomiasis requires trust-building and access to alternatives like clean water.

### Future directions

Achieving WHO’s 2030 schistosomiasis elimination targets require Innovation and integration of the following strategies which are mentioned and outlined strategies towards elimination in East Africa.

### Newer diagnostics and vaccines

Diagnostics should adapt to eliminate appropriate level objectives. The SHERLOCK test’s sensitivity, if that test were adapted for the field, might revolutionize case detection in rural settings^[31]^. Enhanced preservation of Kato–Katz, prolonging slide shelf-life, would be an operational consideration for reference labs both in Tanzania and Uganda^[30]^. Novel diagnostics, such as rapid diagnostics and molecular approaches, are essential for mapping of low-prevalence settings and elimination programs^[23]^. Uganda’s pursuit of CHIMs is promising, as it could speed up the vaccine development process^[33]^. The Sm14 + GLA-SE recombinant vaccine tested in West Africa was safe and highly immunogenic against *S. mansoni* and *S. haematobium*, and warranted further development and advancement to Phase III trials^[37]^. A partially effective vaccine could reduce infections and transmission, addressing praziquantel’s limitation^[3,22]^. The 2023 Uganda Schistosomiasis Symposium highlighted promising vaccine research, though deployment timelines remain uncertain^[33]^.

### One Health integration

There is no alternative to a One Health approach. Rwanda’s example, integrating MDA, WASH, snail control, and improving animal health, shows what can be done^[10]^. Similarly in *S. mansoni* occurs in animals and chimpanzees, and it also encourages veterinary approaches such as animal MDA or environmental sanitation in Uganda against this zoonosis^[17,18,36]^. Machine learning as deployed in Zimbabwe could predict where trachoma would be transmitted in East Africa and direct these interventions well^[29]^. This approach, driven by technology, seems like it belongs in the future, but it requires financing and training to become scalable.

### Climate-adapted strategies

Adaptive control is needed in the face of climate change. Rainfall-triggered, time-limited snail control could lead to a reduction in cercarial emergence, as demonstrated in Tanzania^[14]^. Environmental control, such as modification of irrigation channels to minimize snail habitats, is essential but must be carried out in concert with agricultural considerations^[1,24]^ models suggest snail competitors or predators as nature-based solutions, but field trials are few. In Uganda, GIS and remote sensing methods could assess climate-related risks and efficiently allocate resources^[36]^.

### Programs that are gender-inclusive and community-led

Gender equity is a must. There is a need to focus on female youths as far as MDA is concerned, and teaching programs should consider females’ exposure and cultural perceptions^[7]^. The PHAST model in Tanzania (and Simu ya jamii), through which women can be empowered and poverty reduced, is the paradigm for community-based control^[4]^. Scaling these programs demand local leadership and long-term funding but offers significant socioeconomic benefits.

### Treatment of drug resistance and new drug development

A compound isolated from *Searsia longipes* and *Lannea schimperi* extracts has anti-schistosomal effects in mice, suggesting a possible alternative anti-schistosomal therapy rather than praziquantel^[38]^. The next steps would be to isolate the active chemicals and test toxicity, but the idea of traditional medicine supplementing modern medicine is of interest. New anti-schistosomal drugs recommended in the 2022 WHO guidelines are also needed to mitigate possible praziquantel resistance^[22]^. This dual approach of modern and traditional therapies offers a strategic advantage.

### Policy and investment pledges

Sustained political commitment is vital. Continuing availability of financial resources above and beyond donor cycles is necessary to sustain gains^[35]^. It is crucial for regional policies to align incentives for One Health and climate-adapted strategies from Rwanda^[10]^. Donor and recipient countries, such as the ones that fund Uganda’s control programs, could reduce funding shortfalls^[35]^. Without high level commitment, schistosomiasis risks remaining a neglected disease.

## Conclusion

Schistosomiasis in East Africa reflects both success and challenges. MDA, WASH, and health education have combined to significantly reduce prevalence, with Rwanda and Uganda providing examples of achievable progress. Nevertheless, climate change, diagnosis gaps, gender differences, zoonotic transmissions, and resource limitations remain formidable. Its socioeconomic cost poverty, retarded education, and compromised vaccine response renders it a moral and economic obligation to eliminate.

The future may be in advanced diagnostics, vaccines, the One Health interface, and climate-smart technologies. Community-based gender-inclusive initiatives and alternative therapies are practical ways forward. The tenacity of East African communities and researchers is encouraging, but elimination demands sustained dedication, creativity, and bold action. Progress is evident, but the toughest mile is the last one.

## Data Availability

All relevant data used in this study is provided within the manuscript.

## Research registration unique identifying number (UIN)

This review does not require registry as no protocols was established.
